# The relationship between technostress and work engagement among college physical education teachers: a chain mediation model of job crafting and emotional intelligence

**DOI:** 10.3389/fspor.2026.1938579

**Published:** 2026-09-29

**Authors:** Yihang Deng, Tong Liu, Genzhong Yin, Xudong Jin

**Affiliations:** 1 Physical Culture Institute, Neijiang Normal University, Neijiang, China; 2 School of Sports Training, Chengdu Sport University, Chengdu, China; 3 College of PE & Health Management, Chongqing University of Education, Nanan, Chongqing, China; 4 School of Physical Education, Yunnan Minzu University, Kunming, China

**Keywords:** college physical education teachers, emotional intelligence, job crafting, technostress, work engagement

## Abstract

**Background:**

Technostress refers to a psychological stress state arising from an individual’s inability to cope with the demands or expectations associated with information technology use, and is closely related to teachers’ work attitudes and behavioral performance. However, the psychological mechanism through which technostress is associated with work engagement among college physical education (PE) teachers remains unclear. This study tested a theoretically-based chain mediation model examining the associations among technostress, job crafting, emotional intelligence, and work engagement among college PE teachers, with a particular focus on the theorized sequential mediating roles of job crafting and emotional intelligence.

**Methods:**

The sample consisted of 1,000 college PE teachers from multiple universities across China (52.6% male, 47.4% female; mean age = 38.5 ± 8.7 years). They completed measures of technostress, job crafting, emotional intelligence, and work engagement. Mediation analysis was conducted using SPSS 26.0 with the PROCESS macro (Model 6), and indirect effects were estimated using 5,000 bootstrap resamples.

**Results:**

Technostress was significantly and negatively correlated with work engagement. Regression analyses showed that technostress was found to be a significant negative predictor of job crafting, emotional intelligence, and work engagement, while job crafting and emotional intelligence were positive predictors of work engagement. Mediation analysis indicated that technostress exerted significant indirect effects on work engagement through job crafting alone, emotional intelligence alone, and their sequential pathway. The total indirect effect accounted for 38.04% of the total effect, suggesting a meaningful but not exclusive mediating role of these two variables.

**Conclusion:**

Technostress is negatively associated with work engagement among college PE teachers, with job crafting and emotional intelligence serving both independent and chain mediating roles. These findings highlight that technostress, job crafting, and emotional intelligence represent important psychological pathways associated with work engagement, offering practical implications for designing targeted teacher intervention strategies that are consistent with the theoretical model.

## 1 Introduction

Technostress was initially proposed by Brod (1, 2) as a psychological stress reaction resulting from an individual's inability to effectively adapt to new demands brought by information and communication technology (ICT), characterized by anxiety, fatigue, and decreased work efficiency (2). With the ongoing advancement of educational informatization, online course platforms, intelligent teaching tools, and sports data analysis systems have become deeply integrated into college PE teaching, research, and management. Although technological applications have improved the efficiency of college PE education, research indicates that the manifestations and effects of technostress vary across different occupational groups (3). It is also necessary to distinguish technostress from general work stress. Technostress has a specific technological source—it is a psychological stress state triggered by an imbalance in human–technology interaction, rather than by traditional stressors such as organizational task load or interpersonal relationships (4). This distinction is consistent with broader discussions in the IS literature on technology-driven organizational change (36). However, moderate technological challenges may stimulate teachers’ learning motivation, whereas excessive or inappropriate technological demands may lead to role ambiguity, emotional exhaustion, and increased workload (5). Accordingly, this study focused on college PE teachers’ experiences of technostress rather than general occupational stress.

**Figure 1:**
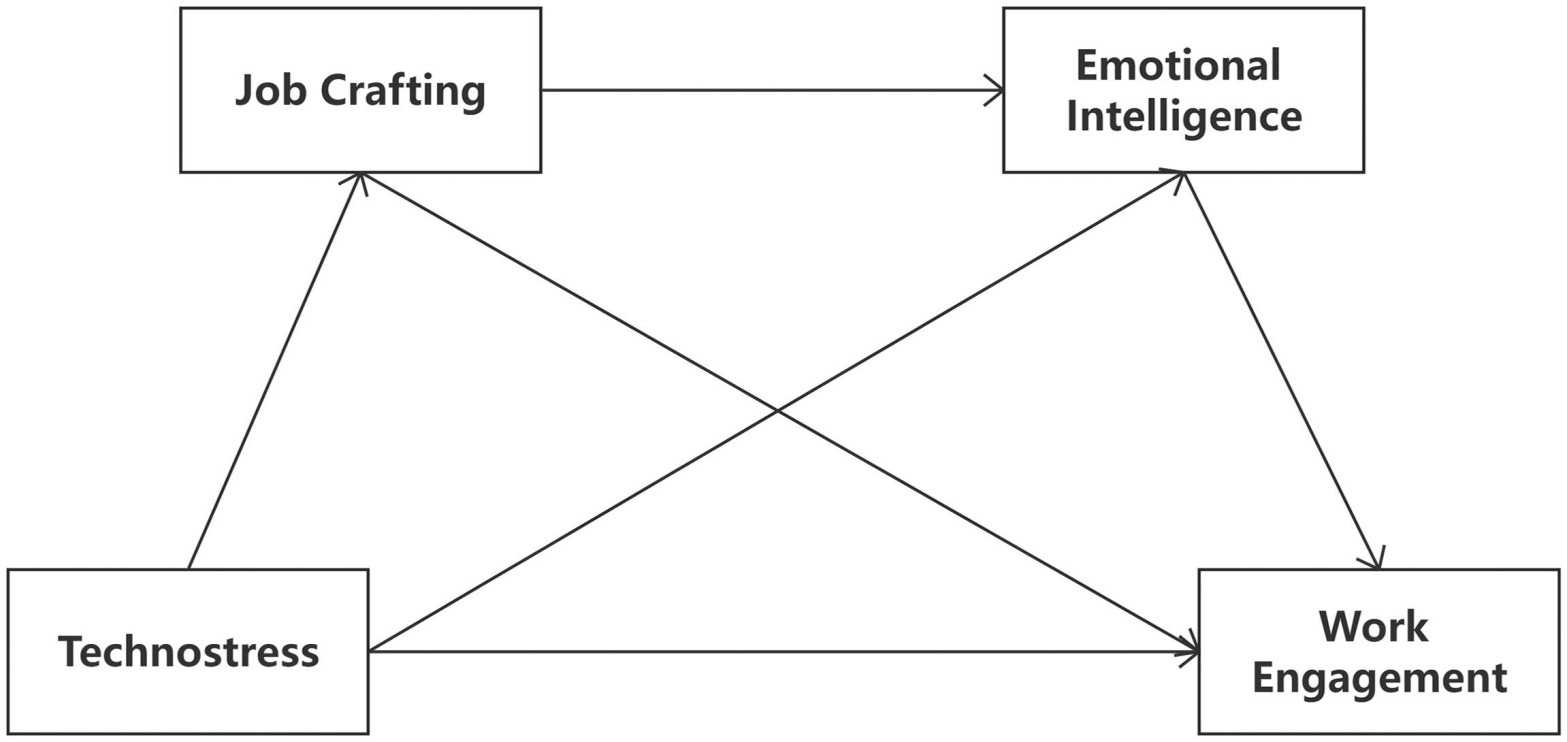
Chain mediation model: technostress and work engagement.

Work engagement refers to a positive, fulfilling, work-related state of mind characterized by vigor, dedication, and absorption (6). As the primary implementers of school PE programs, college PE teachers’ work engagement not only affects the quality of course instruction but also relates to college students’ physical fitness levels and lifelong sports awareness. Teachers with higher work engagement tend to demonstrate greater psychological resilience, work enthusiasm, and concentration, thereby enhancing teaching effectiveness and occupational well-being. However, in the current educational environment, with the rapid iteration of information technology, college PE teachers face difficulties in technological adaptation, insufficient information technology training, and incomplete technical support systems (7), which to some extent undermine their work engagement (8). A survey of university teachers found that during the transformation to information-based teaching, the gap between technological competence and requirements tends to generate techno-anxiety and burnout, with technostress being significantly and negatively correlated with work engagement (9). Therefore, exploring pathways through psychological and behavioral mechanisms to buffer the negative association between technostress and work engagement carries both theoretical and practical significance.

Job crafting refers to a proactive behavioral process through which individuals adjust their work content, work methods, work meaning, and interpersonal relationships to align with their own needs and abilities (10). Related research suggests that job crafting is associated with enhanced sense of professional meaning, work autonomy, and belongingness (11). Positive job crafting helps teachers acquire new resources, improve work experiences, and further enhance their psychological resource reserves and work engagement (12). However, the association between technostress and work engagement is not entirely direct—technostress may be indirectly associated with reduced work engagement through the suppression of teachers’ job crafting behaviors, suggesting that this relationship may be mediated by behavioral-level psychological mechanisms.

Beyond behavioral pathways, emotional resources may also play a significant mediating role between technostress and work engagement. Emotional intelligence refers to an individual's comprehensive ability to recognize, understand, regulate, and utilize emotions in oneself and others (13). As an important emotional resource, emotional intelligence helps individuals cope with stress and maintain positive psychological states. Teachers with higher emotional intelligence are better able to regulate negative emotions and mobilize positive emotions, which may help them maintain higher levels of work engagement when facing technostress (14). The job crafting process may promote the enhancement of individuals’ emotional awareness and regulation abilities. However, existing research has not yet integrated technostress, job crafting, emotional intelligence, and work engagement into a single chain model for systematic examination. That is, technostress may not only be indirectly associated with reduced work engagement through the suppression of job crafting behaviors but may also further amplify this negative association by undermining emotional intelligence.

Research integrating these variables into a chain mediation model and focusing specifically on college PE teachers remains scarce. College PE teachers work in a complex environment: they are not only responsible for routine PE instruction but also organize extracurricular training and competitions, while simultaneously managing increasing information technology demands such as online course development, sports data analysis, and physical fitness monitoring (15). Unlike classroom-based teachers, PE instructors often conduct lessons in gymnasiums or outdoor fields where technology infrastructure is less stable, while simultaneously managing wearable devices, fitness tracking, sports analytics, and digital assessment. These unique demands may make PE teachers particularly vulnerable to technostress. Therefore, examining how technostress is associated with work engagement through the chain mediating mechanism of job crafting and emotional intelligence can not only enrich career development theory for teachers but also provide empirical evidence for targeted interventions.

Although prior research has established the negative association between technostress and work engagement, two theoretical questions remain unanswered. First, it is unclear whether job crafting and emotional intelligence operate sequentially or merely in parallel. Drawing on COR theory, we propose that behavioral withdrawal (reduced job crafting) precedes emotional resource depletion (impaired emotional intelligence), rather than the reverse. Second, it is unclear whether emotional intelligence functions as a stable trait that buffers technostress or as a state-like resource that is depleted under technostress. We adopt the latter perspective, theorizing that technostress consumes the cognitive resources needed to effectively deploy emotional intelligence. These distinctions are central to our proposed chain mediation model.

In summary, this study aimed to test a theory-based sequential mediation model examining the associations between technostress and work engagement among college PE teachers, with a focus on the theorized independent and sequential mediating roles of job crafting and emotional intelligence. Given the cross-sectional design, our analyses test whether the observed pattern is consistent with the theoretical model, rather than establishing causal ordering.

## 2 Literature review and hypotheses

### 2.1 Technostress and work engagement

Related concepts such as job involvement and organizational commitment differ from work engagement. Job involvement emphasizes individuals’ cognitive evaluation of the importance of their work, whereas organizational commitment focuses on individuals’ attachment to and loyalty toward their employing organization (16). Work engagement, by contrast, transcends specific work tasks or organizational belonging, referring to an overall positive affective and cognitive state at work. Research indicates that college PE teachers face challenges including burnout, role pressure, and diminished self-efficacy during their career development, factors that may further undermine their work engagement (17). In the context of rapid educational informatization and the digital transformation of college PE programs, exploring the antecedents and underlying mechanisms of work engagement among college PE teachers carries important practical implications.

According to conservation of resources (COR) theory, individuals tend to strive for acquiring, maintaining, and protecting the resources they value. When individuals face resource loss or an imbalance between resource investment and return, stress ensues, triggering a series of maladaptive occupational behaviors and attitudes (18). Technostress, as a persistent source of resource depletion, consumes teachers’ time and psychological resources that could otherwise be devoted to teaching and professional development (19). Theoretically, this resource loss should be negatively associated with employees’ positive work states, such as work engagement. Specifically, when teachers are compelled to use unfamiliar online teaching platforms, sports data analysis software, or physical fitness monitoring systems, the anxiety and frustration generated during the adaptation process not only directly consume teachers’ cognitive and emotional resources but also diminish their intrinsic interest and value identification with their work, thereby inhibiting work engagement. This reasoning is supported by empirical evidence: technostress negatively predicts teachers’ work engagement, operating indirectly through heightened emotional exhaustion and reduced self-efficacy (20). Based on the above theoretical framework, this study proposed the following hypothesis:

H1: Technostress is negatively associated with work engagement among college PE teachers.

### 2.2 The mediating role of job crafting

Job crafting, as a proactive resource acquisition and management strategy, helps teachers redesign work tasks, expand social support networks, and reframe work meaning, thereby effectively mitigating or preventing the accumulation of work stress (21). However, the relationship between technostress and job crafting is more nuanced than a simple suppressive effect. According to COR theory, when individuals experience sustained resource depletion, they tend to prioritize resource conservation over resource expansion, shifting from approach-oriented to avoidance-oriented strategies (18). In the context of technostress, college PE teachers may selectively reduce certain types of job crafting—particularly task crafting and relational crafting, which require substantial cognitive effort and social energy—while maintaining cognitive crafting as a less resource-intensive means of preserving work meaning (10). Specifically, when PE teachers are forced to spend considerable time troubleshooting technical failures, learning new software, and addressing data security issues, they lack sufficient psychological resources to proactively adjust teaching content to match student interests, strengthen interdisciplinary collaboration, or experiment with optimized instructional methods. The depletion of resources does not entirely eliminate job crafting but rather shifts its nature from expansion-oriented to maintenance-oriented, thereby diminishing its beneficial effects on work engagement. Conversely, if teachers can sustain even minimal levels of proactive job crafting—particularly cognitive reframing of technological challenges as opportunities for professional growth—they may partially preserve their resource reserves and buffer the negative association between technostress and work engagement. Based on this theoretical logic, we proposed the following hypothesis:

H2: Job crafting serves as a mediator in the association between technostress and work engagement among college PE teachers.

### 2.3 The mediating role of emotional intelligence

Based on COR theory, emotional intelligence—as an important personal resource—can promote positive work attitudes and behaviors by enhancing individuals’ emotion regulation abilities, interpersonal skills, and positive emotional experiences (18). However, it is important to distinguish between trait emotional intelligence (a relatively stable capacity) and the effective deployment of emotional intelligence (a state-dependent process). Technostress may not directly “damage” teachers’ inherent emotional intelligence; rather, it depletes the cognitive and attentional resources required to effectively enact emotional intelligence (22). Specifically, when teachers repeatedly experience technical failures, system crashes, and information security threats, the sustained frustration and anxiety triggered by these events consume working memory capacity and attentional control, leaving fewer mental resources available for observing and managing one's own emotions or accurately perceiving others’ emotional states (14). The cognitive overload induced by technostress reduces the psychological bandwidth necessary for self-emotion appraisal, other-emotion appraisal, and emotion regulation—all core components of emotional intelligence as defined by Wong and Law (13). Consequently, although teachers’ baseline emotional intelligence may remain intact, their capacity to mobilize emotional intelligence as a protective resource in the face of technostress is significantly impaired. This diminished functional deployment of emotional intelligence further undermines work engagement: teachers who cannot effectively regulate negative emotions or harness positive emotions during technology-related difficulties are less likely to sustain vigor, dedication, and absorption in their work. Based on this theoretical logic, we proposed the following hypothesis:

H3: Emotional intelligence mediates the relationship between technostress and work engagement among college PE teachers, reflecting the impaired functional deployment of emotional resources under technostress conditions.

### 2.4 The relationship among technostress, job crafting, emotional intelligence, and work engagement

Based on the above theoretical framework, this study proposed that technostress is associated with reduced work engagement through the chain pathway of “resource depletion — behavioral withdrawal (reduced expansion-oriented job crafting) — emotional resource depletion (impaired deployment of emotional intelligence)”. Specifically, technostress, as a persistent source of resource depletion, first shifts teachers’ job crafting from an expansion-oriented to a maintenance-oriented mode — teachers lack sufficient cognitive and emotional resources to proactively broaden teaching tasks, expand social networks, or engage in innovative instructional design. This reduction in expansion-oriented job crafting subsequently impedes the replenishment of emotional resources—when teachers fail to obtain positive work experiences, establish supportive interpersonal relationships, and reframe work meaning through proactive behavioral adjustments, they lose important channels for restoring emotional energy (23). The depletion of emotional resources further undermines teachers’ capacity to effectively deploy emotional intelligence — teachers with diminished emotional resources are less able to regulate negative emotions generated by technostress or to mobilize positive emotions to sustain work enthusiasm and concentration (22). It is crucial to note that while this sequential order is theoretically grounded in COR theory, the present cross-sectional design tests only the statistical associations consistent with this model, not the causal sequence itself. This sequential pathway forms a complete transmission chain from external stressor to behavioral response to emotional state: technostress (external stressor) — reduced expansion-oriented job crafting (behavioral withdrawal) — impaired emotional intelligence deployment (depleted emotional resources) — reduced work engagement (deteriorated occupational psychological state). Moreover, COR theory suggests that this chain may be spirally reinforced: the decline in work engagement further reduces opportunities for teachers to acquire new resources, making it more difficult for them to resume expansion-oriented crafting and emotional resource recovery, thereby amplifying the negative effects of technostress.

Based on the above reasoning, this study proposed the following hypothesis:

H4: Job crafting and emotional intelligence serve as sequential mediators in the association between technostress and work engagement, such that the statistical model predicts an indirect effect through reduced job crafting and impaired emotional intelligence. The sequential order is theoretically proposed rather than empirically established.

Based on the proposed conceptual framework, the following hypotheses were advanced:

1. Technostress is negatively associated with work engagement among college PE teachers.
2. Job crafting mediates the relationship between technostress and work engagement.
3. Emotional intelligence mediates the relationship between technostress and work engagement.
4. Job crafting and emotional intelligence play a chain mediating role between technostress and work engagement.

These hypotheses are grounded in the integrated theoretical framework described above. Testing this chain mediation model will contribute to a deeper understanding of the psychological mechanisms linking technostress to work engagement among college PE teachers.

## 3 Research methods

### 3.1 Research sample

Data were collected using a self-administered questionnaire developed based on existing literature and validated instruments. The questionnaire assessed college PE teachers’ technostress levels, job crafting behaviors, emotional intelligence, and work engagement. Since all participants were Chinese college PE teachers, the questionnaire was administered in Chinese. All scales had been validated previously and demonstrated satisfactory internal consistency and validity. The survey was conducted from February 19 to April 19, 2026, targeting PE teachers from multiple universities across China. A convenience sampling method was adopted. The questionnaire was distributed online via the “Wenjuanxing” platform, supervised by trained research assistants. Before data collection, all assistants familiarized themselves with the questionnaire structure and instructions, and briefed participants on key considerations before they began responding. Participation was entirely voluntary. A total of 1,050 questionnaires were distributed. To ensure data quality, the research team screened and excluded invalid responses based on the following criteria:

Inclusion criteria: (1) currently employed full-time as a PE teacher in a Chinese university; (2) had at least one semester of university-level PE teaching experience; (3) provided voluntary informed consent.
Exclusion criteria: (1) completion time of less than 5 min (the median completion time from a pilot study was 12 min); (2) evidence of patterned responses (e.g., selecting the same option consecutively); (3) failure to correctly answer an embedded attention check item (“Please select ‘moderately agree’ or ’strongly agree’ for this item”); (4) reporting implausible years of teaching or university information (e.g., teaching for over 50 years in a manner inconsistent with reality).

After applying these criteria, 1,000 valid questionnaires were obtained, yielding an effective response rate of 95.2%. Among the valid sample, 52.6% were male (*n* = 526) and 47.4% were female (*n* = 474), with a mean age of 38.5 ± 8.7 years and a mean teaching experience of 12.3 ± 6.4 years. In terms of educational background, 67.2% (*n* = 672) held a master's degree or above, and 32.8% (*n* = 328) held a bachelor's degree. Regarding professional titles, 8.3% (*n* = 83) held senior professional titles, 29.7% (*n* = 297) held associate senior titles, 45.2% (*n* = 452) held intermediate titles, and 16.8% (*n* = 168) held junior titles or below. The sample covered eight provinces across China—Fujian, Sichuan, Heilongjiang, Gansu, Beijing, Guangdong, Hebei, and Anhui, spanning East, Southwest, Northeast, Northwest, North, and South China to ensure broad regional diversity. The present sample was collected independently from our previous investigation of primary and secondary school PE teachers (24). While the prior study covered 10 provinces with 850 participants, the current study specifically targeted college-level educators across 8 provinces, yielding 1,000 valid responses. The two datasets are non-overlapping and represent distinct populations with different occupational stressors and technological contexts. Regarding recruitment, all participants provided informed consent via an online consent form before proceeding. The sample comprised participants from eight provinces: Guangdong (*n* = 154), Beijing (*n* = 143), Sichuan (*n* = 137), Fujian (*n* = 126), Hebei (*n* = 122), Anhui (*n* = 118), Heilongjiang (*n* = 108), and Gansu (*n* = 92). While the sample covers diverse geographic regions, the findings should be generalized to the broader population of Chinese college PE teachers with caution, as the sample was not randomly selected and did not cover all provinces.

### 3.2 Scale design

All instruments were originally developed in English. Following the standard translation and back-translation procedure, the scales were first translated into Chinese by two bilingual researchers independently, and discrepancies were resolved through discussion. A third bilingual researcher then back-translated the Chinese version into English, and the back-translated version was compared with the original to ensure conceptual equivalence. Minor wording adjustments were made to enhance clarity and cultural appropriateness for Chinese college PE teachers.

#### 3.2.1 Technostress

Technostress was measured using the Teachers’ Techno-stress Levels Defining Scale (TTLDS) developed by Çoklar, Efilti, and Şahin (25). This scale is grounded in the theoretical framework of technostress proposed by Brod (2). The scale consists of 28 items rated on a 5-point Likert scale from 1 (“strongly disagree”) to 5 (“strongly agree”), covering five dimensions: learning-teaching process oriented (7 items), profession oriented (6 items), technical issue oriented (6 items), personal oriented (5 items), and social oriented (4 items). It measures teachers’ technostress arising from the integration of ICT into the educational process, with sample items including “I am worried that I won't be able to teach the whole course content because technology use takes time” and “I think technology use increases teachers’ workload.” The total score was calculated by summing item scores across dimensions, with higher scores indicating higher levels of technostress. In this study, the scale demonstrated a Cronbach's alpha coefficient of 0.975 for the total scale. The mean inter-item correlation was 0.56, which—given the scale's multidimensional nature (comprising five theoretically distinct dimensions)—falls within a reasonable range consistent with the high internal consistency and does not suggest problematic item redundancy.

#### 3.2.2 Job crafting

Job crafting was measured using the Job Crafting Scale (JCS) employed in Vogel et al.'s study (23). The scale is grounded in the theoretical framework of job crafting proposed by Wrzesniewski and Dutton (10) and incorporated item designs from Leana et al (26). The scale is unidimensional and consists of four items rated on a 5-point Likert scale from 1 (“almost never”) to 5 (“very frequently”), measuring individuals’ proactive adjustments to their work approaches. Sample items include “introduce new approaches to improve my work,” “change minor work procedures that I think are not productive,” “change the way you do your job to make it easier for yourself,” and “rearrange equipment or furniture in your work environment.” The mean score of the four items was calculated as the job crafting indicator, with higher scores indicating a stronger tendency to engage in job crafting. In this study, the scale demonstrated good internal consistency, with a Cronbach's alpha coefficient of 0.881.

#### 3.2.3 Emotional intelligence

Emotional intelligence was measured using the Wong and Law Emotional Intelligence Scale (WLEIS) developed by Law et al. (27), which is grounded in the theoretical framework of emotional intelligence proposed by Wong and Law (13). The scale consists of 16 items rated on a 7-point Likert scale from 1 (“strongly disagree”) to 7 (“strongly agree”), covering four dimensions: self-emotion appraisal and expression (4 items), others’ emotion appraisal and recognition (4 items), self-emotion management (4 items), and use of emotion (4 items). It measures individuals’ comprehensive emotional abilities, with sample items including “I really understand what I feel,” “I have good control of my own emotions,” “I always tell myself I am a competent person,” and “I am a good observer of others’ emotions.” The mean score of the 16 items was calculated as the emotional intelligence indicator, with higher scores indicating higher levels of emotional intelligence. In this study, the scale demonstrated a Cronbach's alpha coefficient of 0.965 for the total scale. The mean inter-item correlation was 0.56, which—given the scale's multidimensional nature—falls within a reasonable range and does not suggest problematic item redundancy.

#### 3.2.4 Work engagement

Work engagement was measured using the Utrecht Work Engagement Scale-9 (UWES-9), a short version developed by Schaufeli et al. (28), which is grounded in the theoretical framework of work engagement proposed by Schaufeli et al (6). The scale consists of 9 items rated on a 7-point frequency scale from 0 (“never”) to 6 (“always/every day”), covering three dimensions: vigor (3 items), dedication (3 items), and absorption (3 items). It measures individuals’ positive, fulfilling work-related state, with sample items including “At my work, I feel bursting with energy,” “I am enthusiastic about my job,” and “I am immersed in my work.” The mean score of the 9 items was calculated as the work engagement indicator, with higher scores indicating higher levels of work engagement. In this study, the scale demonstrated excellent internal consistency, with a Cronbach's alpha coefficient of 0.950 for the total scale.

### 3.3 Statistical analysis

This study adopted a quantitative approach using cross-sectional survey data to examine how technostress, job crafting, and emotional intelligence are associated with work engagement among college PE teachers. All statistical analyses were performed using SPSS 26.0, the PROCESS macro for SPSS (version 3.4; Model 6), and Excel 2021, following principles of transparency and reproducibility. The scales used in this study had all been previously validated through confirmatory factor analysis (CFA) in Chinese teacher samples (6, 25, 27). Given that the factor structure of each scale has been well established and the primary purpose of this study was to examine relationships among variables rather than scale validation, CFA was not repeated.

First, the collected questionnaires were screened for validity. Of the 1,050 distributed questionnaires, 1,000 were valid, yielding an effective response rate of 95.2%. Second, common method bias was assessed using Harman's single-factor test. All 57 items (28 technostress items, 4 job crafting items, 16 emotional intelligence items, and 9 work engagement items) were entered into an unrotated exploratory factor analysis. The results showed that the first factor accounted for 39.39% of the total variance, below the recommended 40% threshold, indicating that common method bias was not a significant concern in this study (29).

Descriptive statistics and Spearman rank-order correlation coefficients were computed for all study variables (technostress, job crafting, emotional intelligence, and work engagement). Although the Shapiro–Wilk test was significant (*p* < 0.05)—which is common in large samples (*n* = 1,000)—we used Spearman correlations for bivariate analyses to ensure robustness against non-normality, while relying on OLS regression for path coefficients given the central limit theorem. Multiple regression analysis was then performed with technostress, job crafting, and emotional intelligence entered simultaneously as predictors of work engagement, to examine the unique predictive effects of each variable while controlling for the others.

To test the proposed chain mediation model, the PROCESS macro (Model 6) was used with 5,000 bootstrap resamples to generate 95% bias-corrected confidence intervals for the indirect effects. In addition to indirect effects, we report unstandardized and standardized path coefficients, standard errors, model R^2^ values for each endogenous variable, and the proportion of the total effect accounted for by indirect effects as a measure of effect size. This procedure quantified the total, direct, and indirect effects of technostress on work engagement through job crafting and emotional intelligence, assessed both independently and sequentially.

## 4 Results

### 4.1 Sample characteristics

A total of 1,000 valid questionnaires were obtained, yielding an effective response rate of 95.2%. In terms of educational background, 672 participants (67.2%) held a master's degree or above, and 328 (32.8%) held a bachelor's degree. Regarding teaching experience, 189 participants (18.9%) had less than 5 years, 312 (31.2%) had 5 to 10 years, 226 (22.6%) had 11 to 15 years, 86 (8.6%) had 16 to 20 years, and 187 (18.7%) had more than 20 years of teaching experience. The sample included 526 male teachers (52.6%) and 474 female teachers (47.4%). The mean age of the participants was 38.5 years (SD = 8.7), and the mean teaching experience was 12.3 years (SD = 6.4). The sample covered eight provinces across China (Fujian, Sichuan, Heilongjiang, Gansu, Beijing, Guangdong, Hebei, and Anhui), spanning East, Southwest, Northeast, Northwest, North, and South China to ensure broad regional diversity.

### 4.2 Common method bias test

Given the self-report nature of the data collection, Harman's single-factor test was used to assess common method bias. All 57 items were entered into an unrotated exploratory factor analysis, and the results showed that the first factor accounted for 39.39% of the total variance, below the recommended 40% threshold. These results suggest that common method bias was unlikely to be a serious concern in this study.

### 4.3 Descriptive statistics and correlations

Because the Shapiro–Wilk test indicated that all variables deviated from normality (*p* < 0.05), Spearman rank-order correlations were used for correlation analysis. Descriptive statistics and Spearman correlation analyses were conducted for the four variables (technostress, job crafting, emotional intelligence, and work engagement). As shown in Table 1, all variables were significantly correlated with one another:

**Table 1:** Descriptive statistics and correlations of variables.

Variable	Mean	SD	Technostress	Job Crafting	Emotional Intelligence	Work Engagement
Technostress	3.93	0.70	1			
Job Crafting	3.89	0.88	−0.322***	1		
Emotional Intelligence	4.92	1.23	−0.267***	0.459***	1	
Work Engagement	3.91	1.29	−0.492***	0.498***	0.611***	1

*** Indicates: *p* < 0.001.

Technostress was significantly and negatively correlated with work engagement (*ρ* = −0.492, *p* < 0.001); technostress was significantly and negatively correlated with job crafting (*ρ* = −0.322) and with emotional intelligence (*ρ* = −0.267, *p* < 0.001); job crafting was significantly and positively correlated with emotional intelligence (*ρ* = 0.459, *p* < 0.001) and with work engagement (*ρ* = 0.498, *p* < 0.001); and emotional intelligence was significantly and positively correlated with work engagement (*ρ* = 0.611, *p* < 0.001). The correlations among all variables were in line with theoretical expectations, with no extremely high correlations (all absolute correlation coefficients ranged from.267 to.611, none exceeding.85), satisfying the statistical prerequisites for chain mediation analysis.

### 4.4 Mediation effect testing

The proposed chain mediation model is presented in Figure 1. To examine the predictive effects of technostress, job crafting, and emotional intelligence on work engagement, a multiple regression analysis was conducted with all three variables entered simultaneously as predictors. As shown in Table 2, the model significantly predicted work engagement (F = 390.624, *p* < 0.001, R^2^adj = 0.539). Specifically, technostress negatively predicted work engagement (*β* = −0.307, t = −13.386, *p* < 0.001); job crafting positively predicted work engagement (*β* = 0.195, t = 7.775, *p* < 0.001); and emotional intelligence positively predicted work engagement (*β* = 0.457, t = 18.537, *p* < 0.001). The variance inflation factors (VIFs) for all predictors were below 1.36, indicating no multicollinearity concerns.

**Table 2:** R^2^adj denotes the adjusted R^2.^

Variable	Work Engagement				
	*β*	SE	T	F	R^2^adj
Technostress	−0.307	0.023	−13.386***	390.624	0.539
Job Crafting	0.195	0.025	7.775***		
Emotional Intelligence	0.457	0.026	18.537***		

*** Indicates: *p* < 0.001.

It should be noted that Table 2 reports standardized regression coefficients (*β*), whereas Figure 2 reports unstandardized coefficients from the PROCESS model. The two serve different purposes and are not directly comparable.

**Figure 2:**
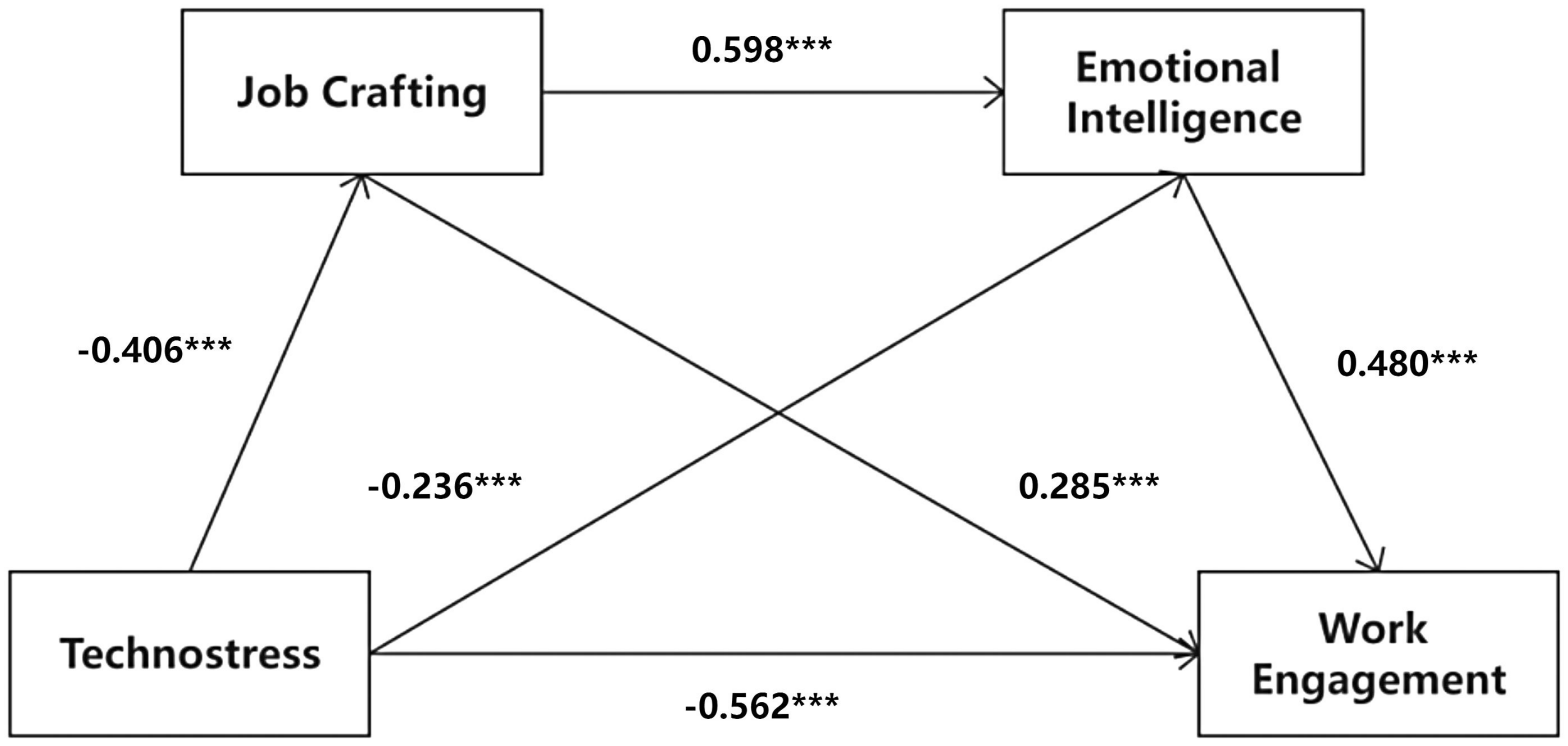
Consolidated path diagram of the full chain mediation model with all path coefficients.

This study examined the chain mediating effects of job crafting and emotional intelligence on the relationship between technostress and work engagement, with technostress as the independent variable, work engagement as the dependent variable, and job crafting and emotional intelligence as mediators. Analyses were performed using the PROCESS macro (version 3.4, Model 6) for SPSS 26.0 to test whether technostress exerted significant mediating and chain mediating effects on work engagement. Hypothesis testing was conducted with PROCESS Model 6, applying 5,000 bootstrap resamples and 95% bias-corrected confidence intervals (CIs) to assess the chain mediation effects.

As shown in Table 3, technostress had a significant negative total effect on work engagement among college PE teachers [total effect = −0.907, *p* < 0.001; 95% CI (−1.006, −0.809)]. This indicates that higher technostress scores were associated with lower levels of work engagement, supporting Hypothesis 1. The relationship between technostress and work engagement was mediated by job crafting [*p* < 0.001; 95% CI (−0.156, −0.081)], indicating that higher technostress was associated with lower work engagement through the suppression of job crafting, thereby supporting Hypothesis 2. Emotional intelligence also mediated the effect of technostress on work engagement [*p* < 0.001; 95% CI (−0.163, −0.068)], indicating that technostress was associated with reduced work engagement through decreased emotional intelligence, which confirmed Hypothesis 3. Finally, job crafting and emotional intelligence sequentially mediated the relationship [*p* < 0.001; 95% CI (−0.147, −0.090)], demonstrating that technostress was associated with lower work engagement through the sequential pathway of inhibiting job crafting and reducing emotional intelligence, thus confirming Hypothesis 4.

**Table 3:** Mediation effect sizes and effect magnitudes.

Path	Effect	Boot SE	BootLLCI	BootULCI	Relative mediation effect	Standardized Effect
Total effect	−0.907***	0.050	−1.006	−0.809	100%	−0.496
Direct effect	−0.562***	0.042	−0.645	−0.480	61.96%	−0.307
Total indirect effect	−0.345***	0.034	−0.415	−0.278	38.04%	−0.189
Indirect effect 1 (JC)	−0.116***	0.019	−0.156	−0.081	12.81%	−0.063
Indirect effect 2 (EI)	−0.113***	0.024	−0.163	−0.068	12.47%	−0.062
Indirect effect 3 (JC→EI)	−0.116***	0.015	−0.147	−0.090	12.81%	−0.064

Indirect effect 1: Technostress — Job Crafting — Work Engagement;.

Indirect effect 2: Technostress — Emotional Intelligence — Work Engagement;.

Indirect effect 3: Technostress — Job Crafting — Emotional Intelligence — Work Engagement.

*** Indicates: *p* < 0.001.

To provide a comprehensive view of the complete model, Figure 2 presents the consolidated path diagram of the full chain mediation model with all path coefficients, allowing readers to examine all hypothesized relationships simultaneously.

## 5 Discussion

### 5.1 Direct association between technostress and work engagement

The results showed a significant negative correlation between technostress and work engagement (*ρ* = −0.492). After controlling for relevant variables and including mediators in the model, the direct association between technostress and work engagement remained significant (*β* = −0.562, *p* < 0.001). Although previous studies have repeatedly shown that higher levels of technostress are associated with lower work engagement, the present findings indicate that technostress alone explains only a modest portion of the variance in work engagement. Thus, while the association is statistically significant, its practical magnitude warrants cautious interpretation. Nevertheless, these results are consistent with the theoretical view that technostress may act as a negative environmental stressor. According to COR theory, such stressors are theorized to deplete psychological resources, which in turn is associated with reduced work engagement. However, this interpretation is theory-driven and should not be equated with empirical evidence of resource depletion *per se*, given the cross-sectional design. This is consistent with previous research showing that technostress is negatively associated with occupational attitudes through negative reactions such as anxiety, fatigue, and decreased efficiency.

This finding suggests that technostress is not only negatively correlated with work engagement at the stress level but may also serve as a signal for college PE teachers—when technological demands exceed individual adaptive capacity, teachers’ work enthusiasm and concentration may be eroded. Unlike teachers who can cope with technological change with relative ease, those experiencing higher levels of technostress tend to adopt defensive strategies to protect remaining psychological resources by reducing investment (18). The observed correlation (*ρ* = −0.492) reflects a moderate-to-strong association between technostress and work engagement. With mediators taken into account, the direct association remained significant (*β* = −0.562), indicating that mechanisms beyond job crafting and emotional intelligence may also contribute to this relationship. This finding not only aligns with the broader literature on technostress but also meaningfully extends our previous work (24). While our prior study demonstrated that job crafting can serve as a resource builder that promotes professional identity through stress reduction and psychological capital accumulation, the present results suggest that technostress diminishes work engagement partly by undermining job crafting as a behavioral resource and impairing the functional deployment of emotional intelligence. This complementary perspective—one study focusing on ‘how to gain‘ positive outcomes and the current study focusing on ‘what is lost‘ under external pressure—offers a more complete picture of how individual behaviors and external stressors jointly shape PE teachers’ occupational well-being.

### 5.2 Mediating effect of job crafting

The indirect effect through job crafting provides statistical support for the theorized behavioral pathway, consistent with the idea that the negative association is partially accounted for by reduced proactive behavioral adjustments, although the magnitude of this single indirect pathway is modest. Its statistical significance, combined with the consistent pattern of findings across the two mediator variables (job crafting and emotional intelligence), reinforces the theoretical plausibility of these behavioral and emotional mechanisms.

Previous research has shown that technostress is negatively associated with job crafting (30), and job crafting in turn is positively associated with work engagement (21). The present study extends this evidence by demonstrating that technostress is not only directly associated with work engagement but may also be indirectly associated with reduced work engagement through the suppression of job crafting, particularly by reducing opportunities for teachers to proactively adjust teaching tasks, expand social support networks, and reframe work cognitions.

This research found that teachers with higher levels of technostress tend to maintain the status quo rather than proactively craft their jobs—they lack sufficient cognitive and emotional resources to make proactive adjustments to their work approaches. Technostress consumes psychological resources that teachers could otherwise use for proactive behavioral adjustment, shifting them from active responders to passive recipients. The reduction in job crafting further is associated with lower work engagement: when teachers are unable to engage in proactive work adjustments, their enthusiasm, dedication, and absorption in work decline. Conversely, if teachers can maintain higher levels of job crafting—even when facing technostress—they may still preserve their resource reserves by proactively adjusting their work, thereby buffering the negative association between technostress and work engagement to some extent.

It should be noted that the job crafting measure used in this study is a unidimensional scale that assesses overall proactive work adjustments rather than distinguishing among task, relational, or cognitive crafting subdimensions. Therefore, our mediation analyses and discussions address job crafting as a general behavioral construct, without claiming to isolate the specific contribution of any particular crafting subtype.

### 5.3 Mediating effect of emotional intelligence

The mediating effect of emotional intelligence was significant (indirect effect = −0.113, accounting for 32.75% of the total indirect effect). This finding is consistent with COR theory, which posits that emotional intelligence (self-emotion appraisal, other-emotion appraisal, self-emotion management, and use of emotion) is positively associated with work attitudes and behaviors by enhancing individuals’ emotion regulation abilities, interpersonal skills, and positive emotional experiences.

Technostress is widely recognized as being negatively associated with individuals’ emotional resources (30), while emotional intelligence serves as a key emotional resource theoretically associated with maintaining stable work engagement among college PE teachers. Consistent with this theoretical perspective, when teachers repeatedly experience technical failures, system crashes, information overload, and other technostress events, sustained frustration and anxiety may be associated with weakened awareness and management of their own emotions. However, this interpretation is based on COR theory and should not be interpreted as empirical evidence of causal depletion. The cognitive overload triggered by technostress may reduce the psychological space available for observing and understanding others’ emotions. Long-term technological adaptation pressure may also diminish teachers’ capacity to use emotions to facilitate constructive behavior. Reduced emotional intelligence may further undermine work engagement: teachers with lower emotional intelligence are more susceptible to emotional dysregulation when facing technical difficulties, making it harder for them to mobilize positive emotional resources to sustain work enthusiasm and concentration, thereby exhibiting lower work engagement. Emotional intelligence, as an emotional resource, plays a notable mediating role in the association between technostress and work engagement, with its indirect effect (−0.113) accounting for a proportion of the total indirect effect comparable to that of job crafting (−0.116).

### 5.4 Chain mediating effect of job crafting and emotional intelligence

Chain mediation analysis revealed that job crafting and emotional intelligence constituted a significant chain mediating pathway between technostress and work engagement [indirect effect = −0.116, 95% CI (−0.147, −0.090)]. This finding is consistent with the theorized sequential pathway. However, causal inference is precluded by the cross-sectional design.

Technostress, as a persistent source of resource depletion, first is associated with reduced teachers’ job crafting behaviors—teachers lack sufficient cognitive and emotional resources to make proactive adjustments to their work. The reduction in job crafting behaviors subsequently is associated with lower emotional intelligence—when teachers are unable to gain positive work experiences and meaningful feedback through proactive work adjustments, their awareness and management of their own and others’ emotions also decline (23). The reduction in emotional intelligence ultimately undermines work engagement—teachers with lower emotional intelligence lack the ability to effectively manage the negative emotions generated by technostress, making it difficult to mobilize positive emotional resources to sustain work enthusiasm and concentration (22). At the same time, COR theory suggests that individual resource loss may exhibit a spiraling effect, whereby initial resource depletion further weakens subsequent resource acquisition and accumulation capacity (31). Accordingly, the decline in work engagement may constrain teachers’ ability to acquire and integrate work resources, making it more difficult for them to sustain proactive work adjustments and effectively regulate emotional resources, thereby further amplifying the negative associations of technostress. This chain mediation is consistent with theoretical frameworks emphasizing the interplay between job crafting and emotional intelligence in shaping occupational attitudes. It also lays a foundation for future research to explore the psychological effects of technostress on teachers’ career development from dynamic and process-oriented perspectives.

Although all indirect effects reached statistical significance, the effect sizes were relatively modest. This phenomenon may be attributable to several factors. First, in chain mediation models, indirect effects are computed as the product of multiple path coefficients, and such products typically yield values smaller than direct effects or single mediation effects (32). Second, the total indirect effect (−0.345) accounted for 38.04% of the total effect (−0.907), indicating that job crafting and emotional intelligence together explain a meaningful proportion of the association between technostress and work engagement. However, this proportional interpretation should be approached with caution: the percentage of total effect mediated is a descriptive index rather than a measure of practical significance, and its magnitude is influenced by both the strength of the indirect pathways and the size of the direct effect retained in the model (33). Third, from an educational administration perspective, even modest effects may carry meaningful implications when applied to large populations of teachers (34). Given that elevated technostress and reduced work engagement among college PE teachers are increasingly prevalent issues in China, interventions targeting the promotion of job crafting and enhancement of emotional intelligence—even if their individual-level effects are relatively small—may still yield non-trivial population-level benefits. Future research should further examine other mediating variables, such as organizational support and self-efficacy, to account for the remaining variance. Taken together, these findings provide empirical support for the proposed chain mediation model, although the modest magnitude of the indirect effects suggests that additional mediating mechanisms not examined in this study (e.g., organizational support, self-efficacy, technology self-confidence) may also contribute to explaining the relationship between technostress and work engagement among college PE teachers.

The observed associations may be particularly relevant to the PE context, where the dual demands of physical instruction and digital monitoring—often in unstable technological environments—may intensify technostress. This suggests that interventions should address the unique technological challenges faced by PE teachers rather than adopting a one-size-fits-all approach.

### 5.5 Limitations and future directions

Several limitations of this study should be acknowledged. First, the cross-sectional design precludes causal inference; the sequential order is a theoretical proposition, not an empirical finding. Alternative models are equally plausible, and all discussions of ‘pathways’ should be interpreted within this statistical and theoretical context. Future research should test the proposed model using longitudinal designs or experimental interventions, for example, by tracking the long-term effects of technostress interventions or job crafting training on teachers’ work engagement.

Second, all variables were measured using self-report instruments, which may be susceptible to recall bias and social desirability effects (29, 35). Although the scales demonstrated adequate reliability and validity, future research could incorporate objective records of technology use (e.g., teaching platform login frequency, number of technical failure reports) or multi-source assessments (e.g., peer evaluations, student feedback, supervisor ratings) to enhance measurement validity. In addition, although Harman's single-factor test suggested that common method bias was not a serious concern (the first factor accounted for 39.39% of the variance, below the 40% threshold), the same-source nature of the data may still inflate the strength of observed associations to some degree.

Third, although the sample covered college PE teachers from eight provinces across China with a relatively balanced gender ratio, these findings may not be fully generalizable to teachers at other educational levels (e.g., primary and secondary schools) or to other occupational groups. Furthermore, the cross-sectional design precludes testing reverse causality. Although our theoretical model assumes that technostress is associated with work engagement, the reverse may also hold: teachers with lower work engagement may be more susceptible to perceiving technostress, or teachers with stronger job crafting abilities may be more inclined to proactively learn new technologies and thereby reduce their technostress perception. Future research should employ longitudinal or cross-lagged panel designs to examine these bidirectional relationships. In addition, the present sample comprised 52.6% male and 47.4% female participants, but no gender-based subgroup analyses were conducted. Although the gender ratio was relatively balanced, male and female teachers may differ in how they cope with technostress, engage in job crafting, express emotional intelligence, and demonstrate work engagement (e.g., female teachers may focus more on relational crafting to buffer technostress, whereas male teachers may emphasize task crafting to address technological challenges). Future research should examine whether gender moderates the proposed chain mediation model. At the same time, variables such as professional title, educational background, teaching experience, and geographic region were not controlled for in the mediation analysis. Although our primary focus was on psychological mechanisms, these demographic and occupational background variables may be related to the observed associations (e.g., teachers with higher professional titles may possess richer teaching experience and technological coping strategies, thereby demonstrating higher work engagement; differences in informatization infrastructure across geographic regions may also influence teachers’ technostress levels). Future research should include them as covariates to improve the precision of estimated associations.

Furthermore, this study did not distinguish between specific dimensions of job crafting (e.g., task crafting, relational crafting, cognitive crafting) or subdimensions of emotional intelligence (self-emotion appraisal and expression, others’ emotion appraisal and recognition, self-emotion management, use of emotion). Different dimensions may play different roles between technostress and work engagement. For example, relational crafting may be more effective than task crafting in alleviating the social isolation associated with technostress, while self-emotion management may be a stronger predictor of work engagement under technostress than others’ emotion appraisal. Future research should examine these dimensional differences in greater depth.

In addition, this study focused only on job crafting and emotional intelligence as mediating variables, but other factors (e.g., organizational support, leadership style, collegial relationships, quality of technology training) may also moderate or mediate the relationship between technostress and work engagement. Future research could explore the roles of these variables, for example, whether the university's information technology support system enhances the positive effects of job crafting, or whether leaders’ technological demonstration behaviors buffer the negative effects of technostress.

Despite these limitations, this study enriches empirical research in the field by systematically clarifying how technostress, job crafting, and emotional intelligence are associated with work engagement among college PE teachers. The findings suggest that alleviating technostress may not only be directly associated with higher work engagement but may also indirectly strengthen it by promoting job crafting and enhancing emotional intelligence. This finding carries practical implications for educational administrators and universities in designing teacher development interventions. Combining technostress management training with job crafting promotion and emotional intelligence cultivation may be an effective approach to enhancing work engagement among college PE teachers. However, technostress is not solely an individual-level phenomenon; it is also shaped by organizational and technological conditions (7). At the organizational level, universities should consider reducing technology workload, improving platform usability, providing accessible technical support, offering ongoing professional development, and ensuring adequate digital infrastructure. At the individual level, targeted training could encourage teachers to proactively adjust their work approaches and enhance their emotional regulation abilities. These complementary strategies—addressing both systemic conditions and individual capacities—would create a more balanced and sustainable approach to mitigating technostress and supporting teacher engagement.

## 6 Conclusion

This study systematically examined the relationship between technostress and work engagement among college PE teachers in China, with a particular focus on the mediating roles of job crafting and emotional intelligence. The findings are consistent with the theoretical model in which technostress is statistically associated with work engagement through the independent and sequential mediating pathways of job crafting and emotional intelligence.

Specifically, technostress was not only directly associated with lower work engagement but was also indirectly associated through its negative correlation with job crafting and its negative correlation with emotional intelligence. Emotional intelligence emerged as a key mediator in the indirect pathway, highlighting its central role in this process. Job crafting, as a behavioral-level resource acquisition pathway, further clarified the psychological mechanism linking technostress to work engagement. Chain mediation analysis further provided patterns consistent with the theoretical proposition that technostress is associated with lower work engagement through reduced job crafting and reduced emotional intelligence.

By integrating behavioral and psychological perspectives, this study extends the existing research framework on the relationship between technostress and work engagement, contributing to a deeper understanding of the mechanisms underlying the formation and erosion of work engagement among college PE teachers. At the practical level, the findings suggest that educational administrators and universities should consider the multifaceted role of technostress management in promoting teachers’ career development. Interventions that combine technostress alleviation with job crafting promotion and emotional intelligence cultivation—particularly strategies that encourage teachers to proactively adjust work tasks to address technological challenges, provide technology training to reduce techno-anxiety, and offer emotion management training to enhance emotional resources—may be especially effective in mitigating the negative effects of technostress and supporting the stability of the college PE teaching workforce.

## Data Availability

The original contributions presented in the study are included in the article/Supplementary Material, further inquiries can be directed to the corresponding author.
